# Radiographic risk factors for degenerative lumbar spondylolisthesis: A comparison with healthy control subjects

**DOI:** 10.3389/fsurg.2022.956696

**Published:** 2022-10-14

**Authors:** Zheng Wang, Yonghao Tian, Chao Li, Donglai Li, Yakubu Ibrahim, Suomao Yuan, Xia Wang, Juan Tang, Shijun Zhang, Lianlei Wang, Xinyu Liu

**Affiliations:** Department of Orthopedics, Qilu Hospital, Cheeloo College of Medicine, Shandong University, Jinan, Shandong, China

**Keywords:** degenerative lumbar spondylolisthesis, disc height, facet joint, paraspinal muscle, radiographic risk factors

## Abstract

**Objective:**

To evaluate the radiologic parameters of degenerative lumbar spondylolisthesis (DLS) and determine the radiographic risk factors for DLS by making comparisons with healthy control subjects.

**Methods:**

Seventy-five patients with L4/5 DLS (Meyerding grade I) and 53 healthy control subjects were analyzed. The L1-S1 disc height index (DHI), L4/5 facet joint angle (FJA), and relative cross-sectional area (RCSA) of paravertebral muscles were measured in both groups. The initial L4/5 DHI (iDHI) before the onset of DLS were estimated based on the L3/4 DHI of the DLS group and DHI of the control group. The sagittal parameters of DLS were also included in this study.

**Results:**

The DHI of L4/5 was lower in the DLS group than in the control group (*P* < 0.05), but the DHI of the L1-L4 segments were much higher than in the control group (*P* < 0.05). The initial L4/5 DHI and FJA of the DLS group were significantly higher than those of the control group (*P* < 0.05). The RCSA of the paravertebral muscles were smaller in the DLS group than in the control group (*P* < 0.05). Binary logistic regression analysis showed that iDHI, FJA, and RCSA of the total paraspinal muscles were risk factors for DLS. The cutoff values for iDHI, FJA, and RCSA were 0.504, 56.968°, and 1.991 respectively. The iDHI was associated with lumbar lordosis (LL), while L4/5 DHI was associated with the RCSA of the multifidus muscle and psoas major muscle (*P* < 0.05).

**Conclusion:**

A large initial lumbar disc height, large FJA, and paravertebral muscle atrophy may be risk factors for DLS.

## Introduction

Spondylolisthesis involves an anterior migration or slip of a vertebra in relation to the next caudal vertebra. Macnab (1) described spondylolisthesis with an intact neural arch — “pseudo-spondylolisthesis”. The term “degenerative spondylolisthesis” was coined by Newman and Stone (2) in 1955, who noted that slippage of vertebrae with an intact neural arch was the result of degenerative arthritis of the lumbar facet joints.

Pope (3) defined spinal instability as displacement of the vertebral bodies due to loss of supportability of the constraining structures, such as the intervertebral discs and facet joints. Vernon-Roberts (4) postulated that degenerative changes of the spine are initiated by structural disorders associated with aging, degeneration, and disc prolapse. Subsequent local or overall disc height decrease leads to forward tilt of the upper vertebral body around the axis of the facet joint, resulting in vertebral instability, facet joint degeneration, osteophyte proliferation, and a series of subsequent changes (5).

The imaging characteristics and risk factors of degenerative lumbar spondylolisthesis (DLS) have been previously investigated. The reported risk factors include female sex (6), lumbar spine degeneration (higher Pfirrmann grade, kyphotic deformity of the sacrum, and facet sclerosis grade) (7–9), more sagittally-oriented facets (10, 11), lumbar lordosis angle, lumbar index (12, 13), shorter transverse process (14), decreased anterior disc height (13), and multifidus muscle atrophy (15). A prospective observation and case-control study with 15-year follow-up in Japan showed a 14% (25/180) incidence of *de novo* DLS during the 15-year period. Progression of the L4 slip (≥3 mm) was observed in 23 participants after 15 years. The significant risk factors for L4 slip progression were identified as age less than 60 years, female sex, lumbar axis sacral distance, facet sagittalization, and existence of slip at baseline (16).

Previous studies have found that the higher and less degenerated have greater intervertebral mobility (17, 18), and disc height decreased at the lesion segment in patients with DLS (13). However, the initial disc height before the lesion has not been studied. The purposes of this study were to investigate the radiographic risk factors of DLS and explore the relationships between intervertebral disc height and other imaging parameters in patients with DLS. We accomplished these by comparing differences in the L1-S1 disc height index (DHI), initial L4/5 disc height index (iDHI), facet joint angle (FJA), and relative cross-sectional area (RCSA) of the paraspinal muscle between patients with and without DLS.

## Materials and methods

### Inclusion and exclusion criteria

**Figure 1:**
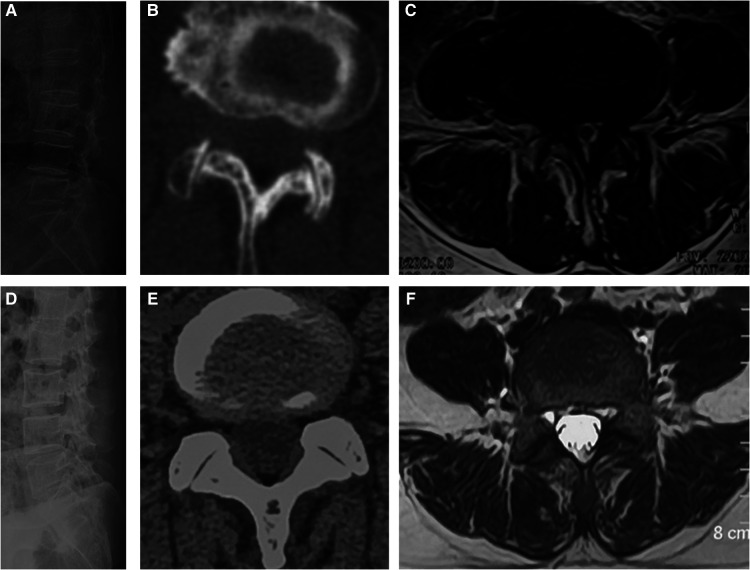
(**A, B and C**) are x-ray, CT and MRI of DLS patients, respectively. (**D, E and F**) are x-ray, CT and MRI of Normal subjects respectively.

This study was approved by the ethics committee of Qilu Hospital of Shandong University and performed in accordance with the Helsinki Declaration. The inclusion criteria were as follows: (1) DLS with Meyerding grade I slippage at L4/5 level; (2) an age between 50 and 70 years; (3) complete pre- and post-operative imaging information (lumbar lateral x-ray, computed tomography, and magnetic resonance imaging data). The exclusion criteria were as follows: (1) lumbar coronal deformity, spine fractures, spine infections, trauma, tumors, and hip and lower extremity disorders; (2) history of previous spinal and/or limb surgery; (3) systemic diseases. The x-ray, CT and MRI of DLS patients and normal subjects were shown in Figure 1.

### General information

The DLS group included 75 patients with L4/5 DLS who received treatment between January 2015 and October 2021 (33 men, 42 women; mean age, 60.4 ± 6.4 years). The control group consisted of 53 participants (23 men, 30 women; mean age, 58.4 ± 5.4 years). The authors counted and compared the BMI (body mass index) and the number of smokers in the two groups.

### Radiographic measurements

#### DHI

**Figure 2:**
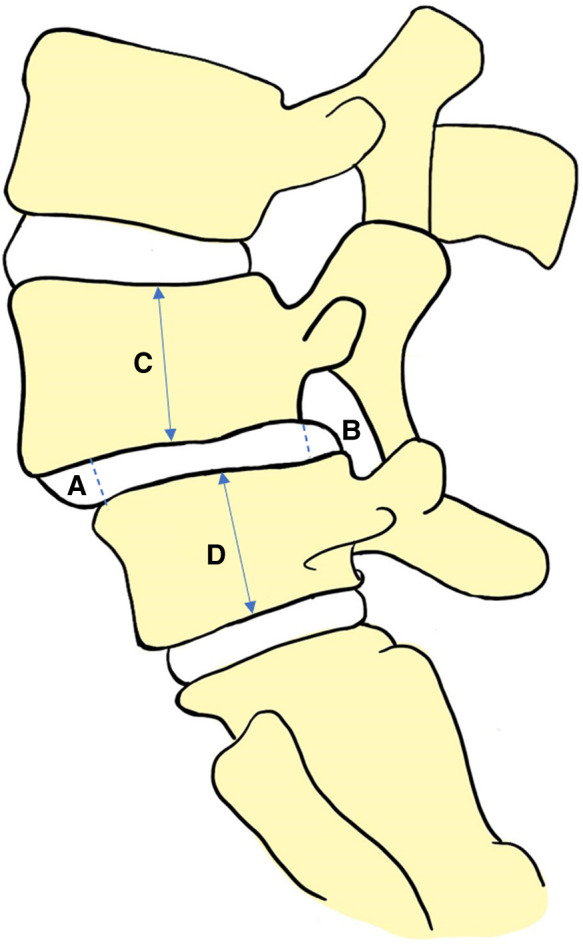
Measurement method of intervertebral disc height index: (A + B)/(C + D).

Sagittal T2-weighted magnetic resonance images of the lumbar spine were used for measurement of the anterior edge height (A) and posterior edge height (B) of the L4/5 intervertebral disc, which was performed using Image J software (NIH Corp., Bethesda, USA). The L4 vertebra height (C) and L5 vertebra height (D) were also measured. DHI was calculated as (A + B)/(C + D), to exclude the influence of individual height and weight differences on intervertebral disc height (Figure 2) (19–21). The DHI of L1/2, L2/3, L3/4, and L5/S1 were measured using the same method.

The L4/5 to L3/4 intervertebral height ratio in Chinese individuals is about 1.14 (22). The ratio of the L4/5 to L3/4 disc height in our control group measured by the method described in the literature (22) was 1.16, which is similar to the published results. Using the L3/4 DHI of the DLS group and the ratio of L3/4 to L4/5 DHI (1.18) of the control group, the initial L4/5 DHI (iDHI) of the DLS group was calculated as L3/4 DHI × 1.18.

#### FJA

**Figure 3:**
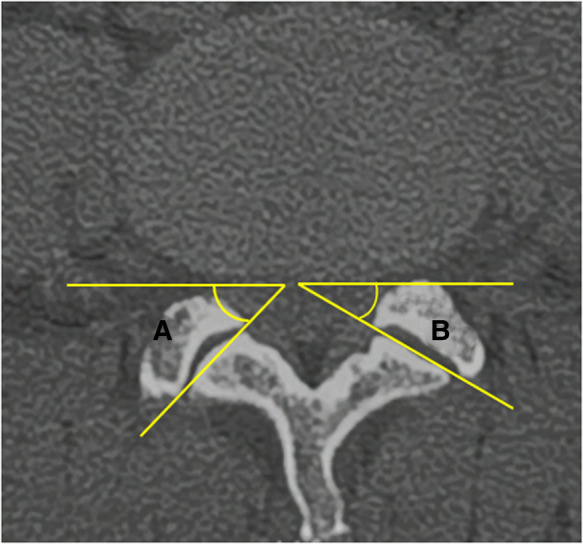
Measurement method of facet joint angle: (A + B)/2.

An axial computed tomography image of the lumbar spine was used for FJA measurement. The middle of the L4/5 vertebral space was identified parallel to the end plate level of the lower edge of L4, and the angle of the connection between the two highest points of the posterior edge of the vertebral body and the connection between the anterior wall of the upper facet and the posterior wall of the lower facet was measured. The right-side FJA was denoted as A and the left as B, and the average angle of both sides was calculated as (A + B)/2 (23) (Figure 3). The average bilateral FJAs were compared between the DLS and control groups. The differences in the average angle were also compared between men and women in the DLS group and the control group.

#### Relative cross-sectional area (RCSA) of paravertebral muscle

**Figure 4:**
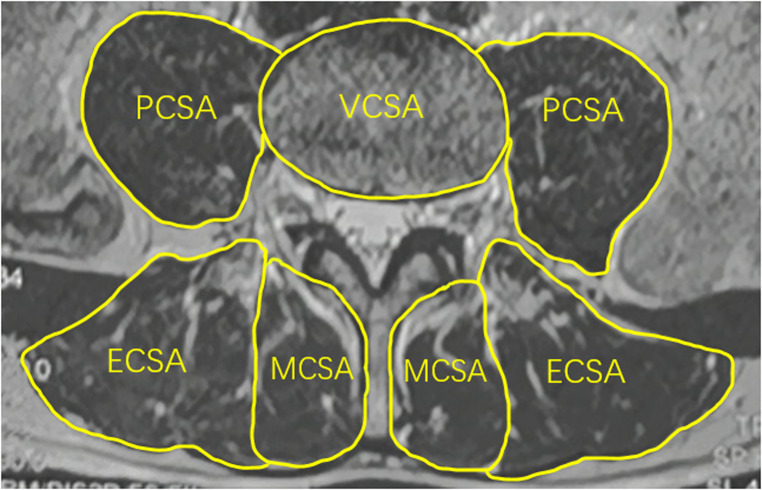
Measurement method of relative cross-sectional area of paraspinal muscles. MCSA, the CSA of multifidus muscle; ECSA, the CSA of erector spinae muscle; PCSA, the CSA of psoas major muscle; VCSA, the CSA of the L4 vertebral body.

An axial T2-weighted magnetic resonance image of the lumbar spine was used to calculate the CSA of paravertebral muscle. The middle of the L4/5 intervertebral space was taken parallel to the end plate of the lower edge of L4. The CSAs of the bilateral psoas major, multifidus, and erector spinae muscles were measured using ImageJ software, and the CSA of each muscle was defined as the boundary of the deep fascia surrounding the innermost muscle. The lower edge of the L4 vertebral body was identified and the CSA of the L4 vertebral body was measured as VCSA. The relative CSA (RCSA) of each paraspinal muscle was calculated and the interactions of height and weight differences on the paraspinal muscle CSA were excluded. The RCSAs were calculated as PCSA/VCSA for psoas major muscle, MCSA/VCSA for multifidus muscle, and ECSA/VCSA for erector spinae muscle (Figure 4). The sum of the RCSAs of the three paraspinal muscles defined the total paraspinal muscle RCSA(T-RCSA) (24). The bilateral average value was taken.

#### Lumbosacral sagittal parameters

**Figure 5:**
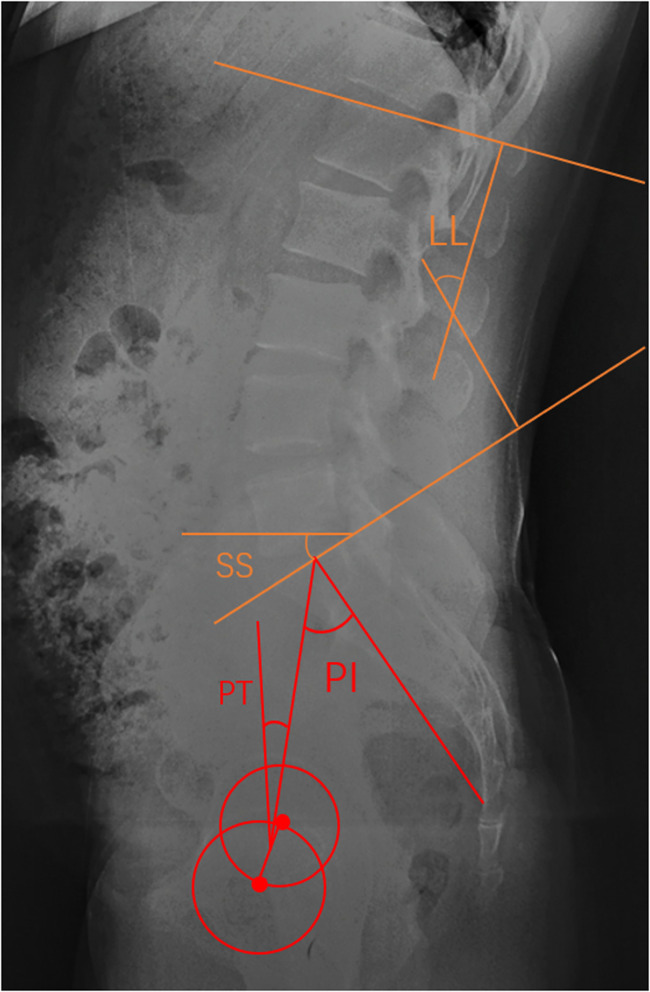
The lumbosacral sagittal parameters, included PI, PT, LL and SS.

All patients underwent a full-spine x-ray examination. Radiological parameters investigated included (1) lumbar lordosis (LL)—the Cobb's angle between the superior endplate of L1 and S1; (2) pelvic incidence (PI)—the angle between the line perpendicular to the sacral plate at its midpoint and the line connecting this point to the axis of the femoral head; (3) pelvic tilt (PT)—the angle between the vertical line and the line connecting the midpoint of the sacral plate to the axis of the femoral head; and (4) sacral slope (SS)—the angle between the sacral plate and the horizontal line. Lordotic angles were noted as positive, and kyphotic ones as negative (25). The measurement methods are shown in Figure 5.

### Statistical analysis

SPSS 22.0 (IBM Corp., Armonk, NY, USA) was used for statistical analysis. Measurement data are expressed as mean ± standard deviation. Independent-samples *t*-tests were used to analyze data with a normal distribution. The imaging parameters of the two groups were analyzed using binary logistic regression with the initial L4/5 DHI, FJA, and total paraspinal muscle RCSA included as independent variables in the binary logistic regression model. Because of a high degree of collinearity between the L4/5 DHI and other data, L4/5 DHI was not included in this model. The odds ratios (ORs), 95% confidence intervals (CIs), and *P*-values of each parameter were calculated using this model. Receiver operating characteristics (ROC) and Youden's index were used to calculate cutoff values for risk factors. In the DLS group, Pearson's correlation was used to determine the correlation between the L4/5 DHI and other parameters, and the correlation between the initial L4/5 DHI and lumbosacral sagittal parameters. A *P* value of <0.05 was considered statistically significant. The inclusion test level was *α *= 0.05.

## Results

The demographic data of the two groups are shown in Table 1. There were no statistically significant differences in age, sex, BMI or number of smokers between the two groups (*P* > 0.05).

**Table 1:** Comparison of results between two groups ($\bar{x}$  ± s).

	DLS group	Control group	*P* value
Age (years)	60.4 ± 6.4	58.4 ± 5.4	0.096
Sex (men: women)	33:42	20:33	0.479
BMI	23.6 ± 4.5	23.4 ± 4.9	0.851
Smokers (%)	15 (20%)	9 (16.7%)	0.666
DHI			
L1/2	0.35 ± 0.05	0.26 ± 0.03	0.000*
L2/3	0.39 ± 0.073	0.29 ± 0.04	0.000*
L3/4	0.44 ± 0.10	0.34 ± 0.06	0.000*
L4/5	0.35 ± 0.13	0.41 ± 0.07	0.017*
Ratio of L4/5 to L3/4	0.80 ± 0.26	1.18 ± 0.15	0.000*
L5/S1	0.40 ± 0.09	0.36 ± 0.09	0.019*
Initial L4/5 DHI	0.53 ± 0.11	0.41 ± 0.07	0.000*
FJA (°)	63.95 ± 9.99	44.64 ± 7.94	0.000*
RCSA			
Multifidus muscle	0.44 ± 0.12	0.58 ± 0.11	0.000*
Erector spine muscle	0.79 ± 0.18	0.90 ± 0.24	0.010*
Psoas major muscle	0.55 ± 0.20	0.71 ± 0.18	0.000*

DLS, degenerative lumbar spondylolisthesis; BMI, body mass index; DHI, disc height index; FJA, Facet joint angle; CSA, Cross sectional area; RCSA, Relative cross sectional area.

The level of statistical significance was set at 0.05.

*P < 0.05.

### DHI

The DHI of L4/5 were lower in the DLS group than in the control group (*P* < 0.05; Table 1), whereas the DHI of L1-4 and L5/S1 segments were higher in the DLS group than in the control group (*P* < 0.05). The ratio of the L4/5 to L3/4 DHI were lower in the DLS group than in the control group (*P* < 0.05). The initial L4/5 DHI was significantly higher in the DLS group (0.53 ± 0.11) than in the control group (0.41 ± 0.07) (*P* < 0.05; Table 1).

### FJA

The L4/5 FJA was higher in the DLS group than in the control group (*P* < 0.05; Table 1), but there was no significant difference between the bilateral FJA in the two groups (*P* < 0.05; Table 2). There was no significant difference between men and women in the DLS group and the control group (*P* < 0.05).

**Table 2:** Comparison of FJA in the two groups ($\bar{x}$ ± s).

	Right side	Left side	*P* value
DLS group	65.19 ± 10.98	62.71 ± 11.29	0.270
Control group	46.01 ± 8.78	43.28 ± 9.03	0.129
	Male	Female	*P* value
DLS group	63.84 ± 10.26	64.03 ± 9.97	0.950
Control group	43.45 ± 8.68	45.65 ± 7.27	0.333

DLS, degenerative lumbar spondylolisthesis; FJA, Facet joint angle.

The level of statistical significance was set at 0.05.

*P < 0.05.

### RCSA of paravertebral muscle

The RCSAs of three paravertebral muscles (multifidus, erector spinae, and psoas major) were smaller in the DLS group than in the control group (all *P* < 0.05; Table 1).

### Lumbosacral sagittal parameters

The mean values of PT, PI, LL, SS, and PI-LL in the DLS group were 23.2 ± 7.7°, 52.8 ± 10.3°, 45.0 ± 12.9°, 29.5 ± 8.5°, and 7.7 ± 11.8°, respectively (Table 3).

**Table 3:** Correlation between initial L4/5 DHI and lumbosacral sagittal parameters.

	Mean value (°)	*R* value	*P* value
PT	23.2 ± 7.7	0.042	0.795
PI	52.8 ± 10.3	0.226	0.162
LL	45.0 ± 12.9	0.361	0.022*
SS	29.5 ± 8.5	0.143	0.236
PI-LL	7.7 ± 11.8	−1.98	0.220

PI, pelvic incidence; PT, pelvic tilt; SS, sacral slope; LL, lumbar lordosis.

*The level of statistical significance was set at 0.05.

### Correlations between the radiographic parameters

**Figure 6:**
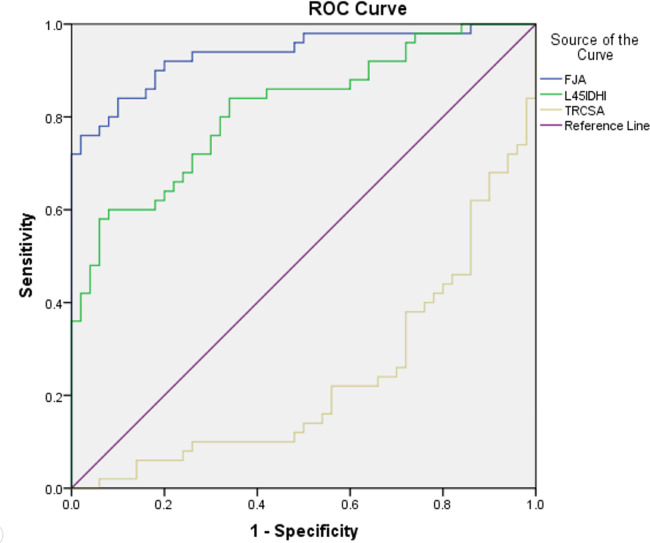
Receiver operating characteristics curve. IDHI: initial L4/5 disc height index, FJA: facet joint angle, TRCSA: relative cross-sectional area of total paraspinal muscle.

The initial L4/5 DHI showed a significant positive correlation with LL (*r* = 0.361, *P* < 0.05; Table 3), but no significant correlation with PI, PT, SS, or PI-LL (*P* > 0.05). L4/5 DHI showed significant positive correlations with L2/3 DHI (*r* = 0.470, *P* < 0.05; Table 4; Figure 6), L3/4 DHI (*r* = 0.529, *P* < 0.05; Figure 6), and L5/S1 DHI (*r* = 0.463, *P* < 0.05; Figure 6) in the DLS group, but no significant correlation with L1/2 DHI (*P* > 0.05). L4/5 DHI showed significant positive correlations with the RCSA of the multifidus muscle (r = 0.390, *P* < 0.05) and psoas major muscle (*r* = 0.294, *P* < 0.05; Table 4, Figure 6), but no significant correlation with the RCSA of the erector spinae muscle (*P* > 0.05).

**Table 4:** Correlation between L4/5 DHI and other imaging parameters.

	*R* value	*P* value
DHI		
L1/2	0.017	0.907
L2/3	0.470	0.001*
L3/4	0.529	0.000*
L5/S1	0.463	0.001*
FJA	0.028	0.849
RCSA		
Multifidus muscle	0.390	0.005*
Erector spine muscle	0.187	0.194
Psoas major muscle	0.294	0.038*

DHI, disc height index; FJA, facet joint angle; RCSA, relative cross sectional area of total paraspinal muscle.

The level of statistical significance was set at 0.05.

*P < 0.05.

### Radiographic risk factors for DLS

The risk factors for DLS were initial L4/5 DHI (OR = 1.443, 95% CI = 1.081–1.927, *P* = 0.013), FJA (OR = 1.845, 95% CI = 1.210–2.813, *P* = 0.004), and RCSA of the total paravertebral muscle (OR = 0.495, 95% CI = 0.289–0.847, *P* = 0.010; Table 5).

**Table 5:** Logistic regression analysis between 2 groups.

	OR	95% CI		*P* value
Initial L4/5 DHI	1.443	1.081–1.927		0.013*
FJA	1.845	1.210–2.813		0.004*
T-RCSA	0.495	0.289–0.847		0.010*
ROC curve	AUC	Standard error	*P* value	95% CI
Initial L4/5 DHI	0.812	0.042	0.000*	0.729–0.895
FJA	0.936	0.025	0.000*	0.888–0.984
T-RCSA	0.236	0.048	0.000*	0.142–0.330
Cutoff value and Yuden index	Cutoff value	Sensitivity	Specificity	Yuden index
Initial L4/5 DHI	0.504	58.0%	94.0%	0.52
FJA	56.968	76.0%	98.0%	0.74
T-RCSA	1.991	70.0%	76.0%	0.46

DHI, disc height index; FJA, facet joint angle; T-RCSA, relative cross-sectional area of total paraspinal muscle.

The level of statistical significance was set at 0.05.

*P < 0.05.

### ROC curves and Youden's indices of the risk factors

**Figure 7:**
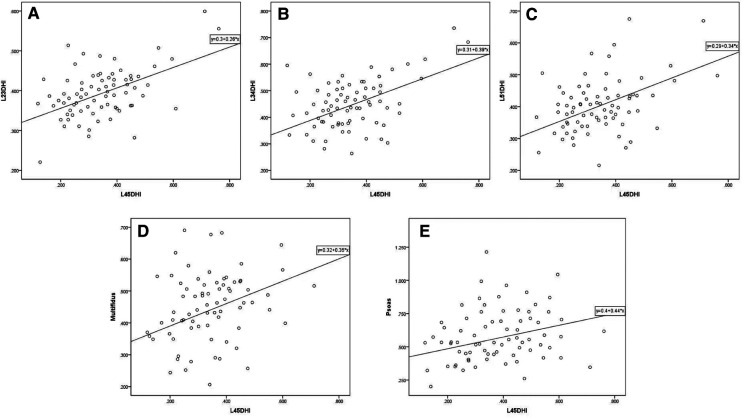
Scatter plot of L4/5 DHI and other imaging parameters in DLS group. (**A**, **B**, **C**) L4/5 DHI showed significant positive correlations with L2/3 DHI (*r* = 0.470, *P* < 0.05), L3/4 DHI (*r* = 0.529, *P* < 0.05), and L5/S1 DHI (*r* = 0.463, *P* < 0.05) in the DLS group. (**D**, **E**) L4/5 DHI showed significant positive correlations with the RCSA of the multifidus muscle (*r* = 0.390, *P* < 0.05) and psoas major muscle (*r* = 0.294, *P* < 0.05). DHI: disc height index, Multifidus: relative cross-sectional area of multifidus muscle, Psoas: relative cross-sectional area of psoas major muscle.

For the prediction of DLS, the area under the ROC curve (AUC) of the initial L4/5 DHI was 0.812 (standard error, 0.042; *P* = 0.000; 95% CI = 0.729–0.895; Table 5, Figure 7). With a cutoff value of 0.504, the sensitivity, specificity, and Youden's index of the iDHI were 58.0%, 94.0%, and 0.52 respectively, (Table 5). The AUC of FJA was 0.936 (standard error, 0.025; *P* = 0.000; 95% CI = 0.888–0.984; Table 5, Figure 7), and with a cutoff value of 56.968, the sensitivity, specificity, and Youden's index were 76.0%, 98.0%, and 0.74, respectively (Table 5).

The AUC of the total paraspinal muscle RCSA was 0.236 (standard error, 0.048; *P* = 0.000; 95% CI = 0.142–0.330). After adjusting the test direction, the AUC was 0.764 and the cutoff value 1.991, providing sensitivity, specificity, and Youden's index of 70.0%, 76.0%, and 0.46, respectively.

## Discussion

Our study showed that a large initial lumbar DHI, a large FJA, and paravertebral muscle atrophy are risk factors for DLS.

Similar to previous studies (14), we found that the DHI of L4/5 was much lower in patients with DLS than in healthy control subjects. Berlemann (7) found that the severity of lumbar spondylolisthesis was significantly negatively correlated with disc height after lesion occurrence, and was associated with the sagittal alignment of the L4/5 facet joint. The decreased disc height and volume were mainly caused by disc degeneration (26), with DLS patients having a high degree of disc degeneration (10). To date, most studies only focused on the disc height of the affected level, ignoring the disc height of upper and lower segments. In our clinical practice, we noted that the adjacent disc height is usually high in patients with DLS, and therefore wondered whether the initial intervertebral height might be related to the occurrence of DLS.

In healthy Chinese individuals, the L4/5 to L3/4 intervertebral height ratio is about 1.14 (22). The ratio of the L4/5 to L3/4 disc height in our control group measured by the method described in the literature (22) was 1.16, which is similar to the published results. Thus, we consider our estimates of initial L4/5 DHI to be reliable and suitable for statistical analysis. Our estimated initial L4/5 DHI in the DLS group was significantly higher than the measured L4/5 DHI in the control group, and L1-4 and L5/S1 DHIs were also significantly higher in the DLS group than in the control group. Logistic regression showed that patients with a large initial L4/5 DHI were more prone to DLS, with an optimal cutoff value of 0.505. Therefore, the authors stipulated that this initial higher intervertebral height at the affected level may play an important role in slip progression in DLS, and act as a risk factor for DLS. When the spine is over-extended and over-flexed, a higher disc height is associated with lower disc stiffness and a greater risk of deformation, which may be related to disc geometry, calcification, or degenerative changes (27). As a result, the posterior column of the spine bears a greater load during activity, and the small joint capsule and ligament will withstand greater stretch tension (28). Many authors have measured spinal mobility with respect to the effect of age and disc degeneration (29). Lumbar mobility is determined by the geometry and material properties of the intervertebral structures, the higher discs and less degenerated dics have greater intervertebral mobility (17). Studies have shown that discs can undergo rapid deformation in response to changes in pressure, and the rapid deformation was associated with nucleus pulposus and endplate flow. Thus, high discs with greater mobility and deformability have more intense nucleus pulposus flow and are more susceptible to disc degeneration (18). When the discs and facet joints are unstable, the lumbar spine is subjected to shear forces, resulting in grade 1 DLS (30). As the disc degenerates, the disc height decreases, the supporting pressure effect and spinal flexibility decreases as a result (17).

Among lumbosacral sagittal parameters, a large LL and PI were found to be significant predictors of L4 DLS by some researchers (31), with a cutoff value of 45.0° for LL, the same value as the mean LL of the DLS patients in the current study. We also analyzed the initial L4/5 DHI and lumbosacral sagittal parameters and found a significant positive correlation between the initial L4/5 DHI and LL. Therefore, the higher disc height is associated with lumbar instability. The LL of patients with DLS is larger than that of age-matched subjects without DLS (16, 31). This larger LL causes L4 to become the apex of LL and applies greater shear force that increases spinal instability, causing DLS.

Many studies showed that sagittally-oriented facets at L4/5 may be a risk factor for DLS (32). Coronal facet surfaces can withstand greater shear forces than sagittal facet surfaces. Therefore, the intervertebral discs and capsular ligaments of sagittal facet joints are more susceptible to further damage due to anterior-posterior shear forces. In our study, the FJA of patients with DLS was significantly larger than that of control subjects of the same age. The cutoff value for the angle as a risk factor was 57°. Abnormal morphology of the lumbar articular processes is a predisposing factor for the development of DLS. Although many studies have revealed sagittal deviation of the facet joints in DLS, it is difficult to demonstrate that this is a preexisting factor for lumbar spondylolisthesis. Our study also showed no correlation between FJA and DHI. Moreover, joint asymmetry can lead to uneven stresses on the small joints and uneven distribution of pressure and biomechanical forces on the intervertebral discs, which can eventually lead to DLS. Current studies report the incidence of joint asymmetry to be between 40% and 70%, with L4/5 being the most commonly affected segment (33). Some authors have found that joint asymmetry is significantly associated with DLS (11, 34). Devanand found that facet angle sagittalization was significantly associated with the L5-S1 level in men and the L4-5 level in women (35). However, we did not find a significant difference in bilateral FJA between the DLS and control group, and no significant difference was found between men and women in either the DLS or control group.

The paraspinal muscles, including the multifidus, erector spinae, and psoas major, are very important for maintaining spine stability and lumbar lordosis (36). In patients with DLS, both the degree of multifidus atrophy and the T2 signal intensity are increased, suggesting that fat infiltration reduces muscle strength and may lead to instability of adjacent vertebrae (15). In this study, the RCSA of the multifidus muscle in the affected segment was significantly smaller than that in the control group, suggesting that multifidus muscle atrophy occurred in patients with spondylolisthesis. Furthermore, atrophy of the erector spinae and psoas major muscles was also observed in the DLS group, and our statistical analysis showed a decreased total paraspinal muscle area (with a cutoff value of 1.991 for prediction of DLS), which facilitated the occurrence of DLS.

In patients with LDH or spinal stenosis, the degree of disc degeneration is positively correlated with paraspinal atrophy (36, 37), and a smaller intervertebral space in DLS is associated with a greater degree of vertebral slippage (7, 38), resulting in more severe squeezing or pulling of nerve roots. When the dorsal ramus nerve is covered and compressed by scar tissue after surgery, the local paraspinal muscles of the corresponding segment will undergo denervation atrophy (39). Tamai et al. (40) found that multifidus atrophy was related to the severity of lumbar spondylolisthesis and that intervertebral disc degeneration could interact with paravertebral muscle fat infiltration. Our results also indicate that a lower DH is associated with paraspinal muscle atrophy.

The current study also has several limitations. First, this study is a retrospective study, there are certain limitations in the study of the pathogenesis of degenerative lumbar spondylolisthesis and the order of changes in imaging parameters. More prospective studies and basic studies such as physiology, pathology, anatomy, cytology, and ergonomics may be needed for further explorations. Second, the small sample size significantly limits the generalizability of the results to a wider population. Thirdly, as the study has been conducted on a Chinese population, it is possible that the external validity of the results may not be applicable to other populations with different anthropometric characteristics.

## Conclusions

A large initial lumbar disc height, large FJA, and paravertebral muscle atrophy may be risk factors for DLS.

## Data Availability

The raw data supporting the conclusions of this article will be made available by the authors, without undue reservation.
